# Far Lateral Tubular Decompression: A Case Series Studying One and Two Year Outcomes with Predictors of Failure

**DOI:** 10.7759/cureus.5133

**Published:** 2019-07-13

**Authors:** Ziyad O Knio, Wesley Hsu, Alejandro Marquez-Lara, Tianyi D Luo, John M St.Angelo, Suman Medda, Tadhg J O'Gara

**Affiliations:** 1 Department of Orthopaedic Surgery, Wake Forest School of Medicine, Winston-Salem, USA; 2 Department of Neurosurgery, Wake Forest School of Medicine, Winston-Salem, USA; 3 Department of Orthopaedic Surgery, Wake Forest Baptist Medical Center, Winston-Salem, USA

**Keywords:** lumbar nerve decompression, tubular decompression, foraminal stenosis, minimally invasive surgery, disability, back pain, leg pain

## Abstract

Introduction

The optimal surgical treatment of isolated lumbar foraminal stenosis has not been defined. Minimally invasive decompression of the foramen from a far lateral tubular decompression (FLTD) approach has been shown to not only have minimal morbidity but also highly variable success rates at short-term follow-up. It is important to quantify improvement and define the demographic and radiographic parameters that predict failure in this promising, minimally invasive surgical technique. This study investigates pain and disability score improvement following FLTD at 12 and 24 months and investigates associations with failure.

Methods

All patients who underwent lumbar FLTD by a single surgeon at a single institution from September 2015 to January 2018 were included in this prospective case series. Visual analog scale (VAS) for back pain and leg pain and Oswestry Disability Index (ODI) were collected preoperatively and at the 12- and 24- month follow-ups. Outcomes between visits were fitted to a linear mixed-effects model. The univariate analysis investigated demographic, radiographic, and operative associations with subsequent open revision.

Results

A total of 42 patients were included in this study. Back pain (VAS 5.84 to 3.32, p<0.001), leg pain (VAS 7.33 to 2.71, p<0.001), and ODI (48.97 to 28.50, p<0.001) demonstrated significant improvements at the 12-month follow-up. Back pain (VAS 3.71, p=0.004), leg pain (VAS 3.04, p<0.001), and ODI (30.63, p<0.001) improvements were maintained at 24-month follow-up. Four patients (9.5%) required subsequent open revision. Subsequent open revision was associated with prior spine surgery (RR=2.85 (2.07-3.63), p=0.045) and scoliosis ≥10° (RR=6.33 (4.87-7.80), p=0.013).

Conclusion

Back pain, leg pain, and ODI showed significant improvement postoperatively. Improvement is maintained at two years. Prior spine surgery and scoliosis ≥ 10° may be relative contraindications to FLTD.

## Introduction

Foraminal stenosis causing radicular leg pain affects 8%-11% of lumbar spinal stenosis patients [1]. Unlike the more common causes of lumbar spinal stenosis in the central canal and lateral recesses, stenosis at the neuroforamen necessitates targeted surgical approaches due to its unique anatomy [2]. The optimal surgical approach for foraminal decompression continues to attract much debate. Symptomatic foraminal stenosis often exists concomitantly with the more common central and lateral recess varieties and, in these situations, the surgical approach must be tailored to address both anatomic locations. In situations where the foramen is the only location of pathology, a minimally invasive, outside-in approach can decompress the nerve root with minimal dissection and morbidity [3-6]. In the published literature, there are a few technical variants to this outside-in approach with a reported success rate ranging from 56%-88% in patients with radicular symptoms from neuroforaminal stenosis. Furthermore, factors associated with treatment failure include the L5-S1 level, higher coronal disc wedge angles, and higher segmental sagittal lordosis [4-5,7]. Despite great advances in minimally invasive surgical (MIS) techniques, there is limited data on the long-term outcomes associated with a far lateral MIS decompression and factors associated with symptomatic relief.

The purpose of this study was to investigate one- and two-year outcomes following far lateral tubular decompressions (FLTD) of the lumbar neuroforamen, as well as the patient and surgical factors associated with failure. We hypothesized that patients undergoing FLTD would demonstrate a significant improvement from presurgery to the 12-month follow-up and maintain the improvement at the 24-month follow-up.

## Materials and methods

This study was conducted at a single academic medical center with prospective data collection beginning in September 2015. Outcomes were collected at 12-month intervals as the standard of care. All aspects of the study were approved by the institutional review board. Consecutive patients with symptomatic degenerative lumbar foraminal stenosis who underwent FLTD by the senior author were included in this study.

Patient selection

Inclusion criteria for this study were patients ≥18 years of age with symptomatic foraminal stenosis who underwent elective microdecompression of the lumbar spine. Patient selection for surgery was based on the preoperative evaluation by the senior author and the persistence of neurologic deficits after at least 90 days of conservative non-operative treatment (e.g., activity modification, non-steroidal anti-inflammatory drugs, physical therapy, and/or exercises). Selection criteria included the absence of a lytic spondylolisthesis at the affected level. Degenerative spondylolisthesis was not a contraindication to this approach. Magnetic resonance imaging (MRI) selection criteria included radicular symptoms that anatomically correlated with MRI evidence of nerve root compression in the foramen. The foramen size was not measured; instead, the nerve root was visualized on axial and sagittal T2 imaging throughout its course: If the root itself was compressed in the mid-zone or exit zone of the neuroforamen, the pathoanatomy was deemed amenable to far-lateral decompression. If significant lateral recess stenosis also involved this nerve root, patients were treated with a dual surgical approach: a tubular decompression laminotomy of the lateral recess combined with the far lateral approach.

Surgical technique

**Figure 1:**
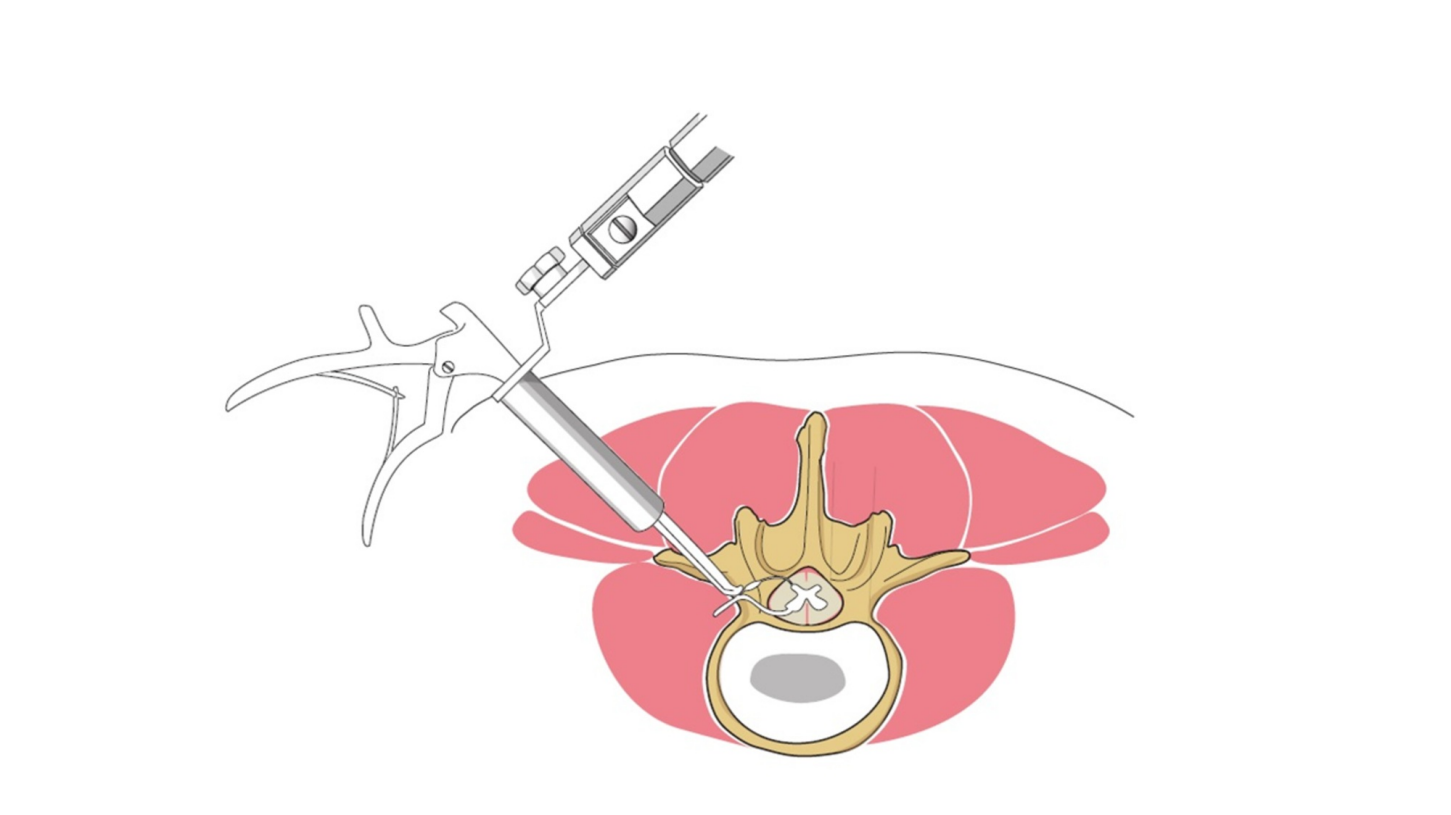
Far lateral tubular decompression approach.

**Figure 2:**
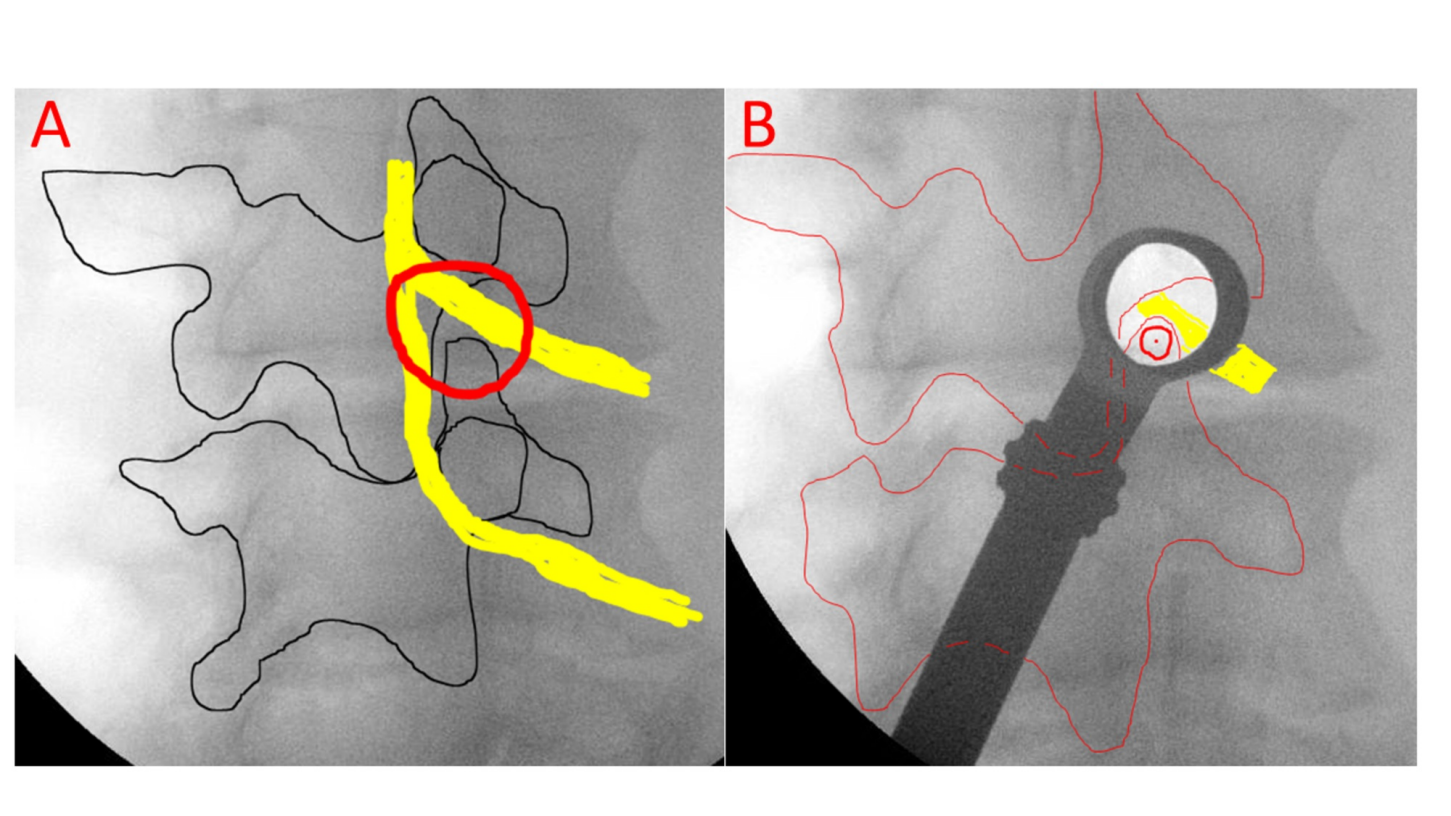
Intraoperative imaging with landmarks and illustrated nerves overlaid (a) and tubular retractor in place (b).

The surgical approach used was slightly different than the previously published techniques in that oblique fluoroscopy was used to approach the neuroforamen from a more lateral approach, sometimes up to 9 cm off midline [5,8]. This oblique approach permitted decompression of the foramen medial to the pedicle with minimal resection of the pars interarticularis (Figure 1). A surgical microscope was used in all cases. All patients were placed prone on a spine table and oblique fluoroscopy was used to dock the METRx™ tubular retractor (Medtronic, Dublin, Ireland) to the superior articular process (Figure 2). The foramen was then entered by burring the superolateral portion of the inferior vertebra’s superior facet. Burring or resection of the pars interarticularis was not needed to gain access medial to the pedicle. With this technique, the skin incision ranged from 5-9 cm off midline.

Data collection

Visual analog scale (VAS) for back and leg pain and Oswestry Disability Index (ODI) were collected preoperatively and at 12-month intervals as the standard of care for all patients undergoing lumbar nerve decompression. Baseline scores were collected during preoperative office visits. Postoperative outcomes were either collected during follow-up office visits or via a standardized telephone script.

ODI is a composite score of the responses to 10 independent questions [9]. In cases where patients chose not to answer one or more questions, the score was computed from the available responses and scaled by a factor of 10 / (10 - # of missing responses). No modifications were made to the raw score recorded for VAS.

Patient demographics (age, gender, BMI, current smoker, and diabetes diagnosis) and operative details were retrieved from the institutional electronic medical records. The operative time reported is the operative time per level; i.e. two-level decompression operative times were reduced by a factor of two. Spondylolisthesis, scoliosis, coronal wedge angle, and segmental lordosis angle measures from preoperative standing lumbar spine X-rays were assessed by a single reviewer. The coronal disc wedge angle of the operated segment was recorded as per the method used by Haimoto: “coronal angle between the line parallel to the lower endplate of the superior vertebra and the line parallel to the upper endplate of inferior vertebra” [4]. A positive angle referred to the operated side being closed down and on the concavity of the curve. The segmental lordosis angle at the operated level was recorded. At L5-S1, this corresponded to the angle created by lines through the superior endplates of L5 and S1. At the other lumbar levels, this corresponded to the angle created by lines through the superior endplate of the superior vertebra and the inferior endplate of the inferior vertebra.

Statistical methods

All computations and statistical analyses were performed in R (R Core Team, Vienna, Austria). A linear mixed-effects model was fitted to outcomes and preoperative scores, controlling for differences between patients. Pairwise differences were investigated with a post-hoc Tukey test for multiple comparisons. This methodology allowed for an investigation of whether pain and disability scores demonstrated improvement from pre-surgery to 12-month follow-up, whether the improvement was maintained at 24-month follow-up, and whether there was any deterioration between 12-month and 24-month follow-up. A p-value of 0.05 was considered significant for outcomes improvement.

Patient demographic, radiographic, and operative factors were compared between those who did and did not require subsequent open revision. These comparisons were quantified with a Mann-Whitney U test for continuous variables and with Pearson’s Chi-Square Test for categorical variables.

## Results

A total of 42 consecutive patients who underwent FLTD were included in the study. Given the prospective nature of the data collection, 28 patients were eligible for two-year outcomes inclusion and 14 patients were eligible for only one-year outcomes inclusion. Four patients (9.5%) underwent multi-level FLTD. Eight patients (19.0%) had simultaneous discectomy. Two patients (4.8%) required a dual surgical approach (tubular decompression laminotomy of the lateral recess combined with the far-lateral approach) to address lateral recess stenosis. A summary of patient characteristics is presented in Table 1.

**Table 1:** Patient characteristics of FLTD cases. BMI: body mass index, FLTD: far lateral tubular decompression, p: proportion, SD: standard deviation

Variable	Mean ± SD	n
	p (%)	
	n=42	
Age (years)	67.50 ± 12.90	42
Wedge angle (°)	2.15 ± 2.39	34
Segmental lordosis (°)	21.41 ± 7.82	34
Operative time (minutes)	90.09 ± 34.28	41
Length of stay (days)	0.76 ± 1.15	42
Gender (male)	25/42 (59.5%)	42
BMI>30	19/40 (47.5%)	40
Current smoker	4/42 (9.5%)	42
Diabetes	8/42 (19.0%)	42
Synovial cyst	3/42 (7.1%)	42
Multiple levels	4/42 (9.5%)	42
Prior spine surgery	13/42 (31.0%)	42
Disc procedure	8/42 (19.0%)	42
Laminotomy	2/42 (4.8%)	42
Spondylolisthesis ≥ 4 mm	6/42 (14.3%)	42
Scoliosis ≥ 10°	5/42 (11.9%)	42

**Figure 3:**
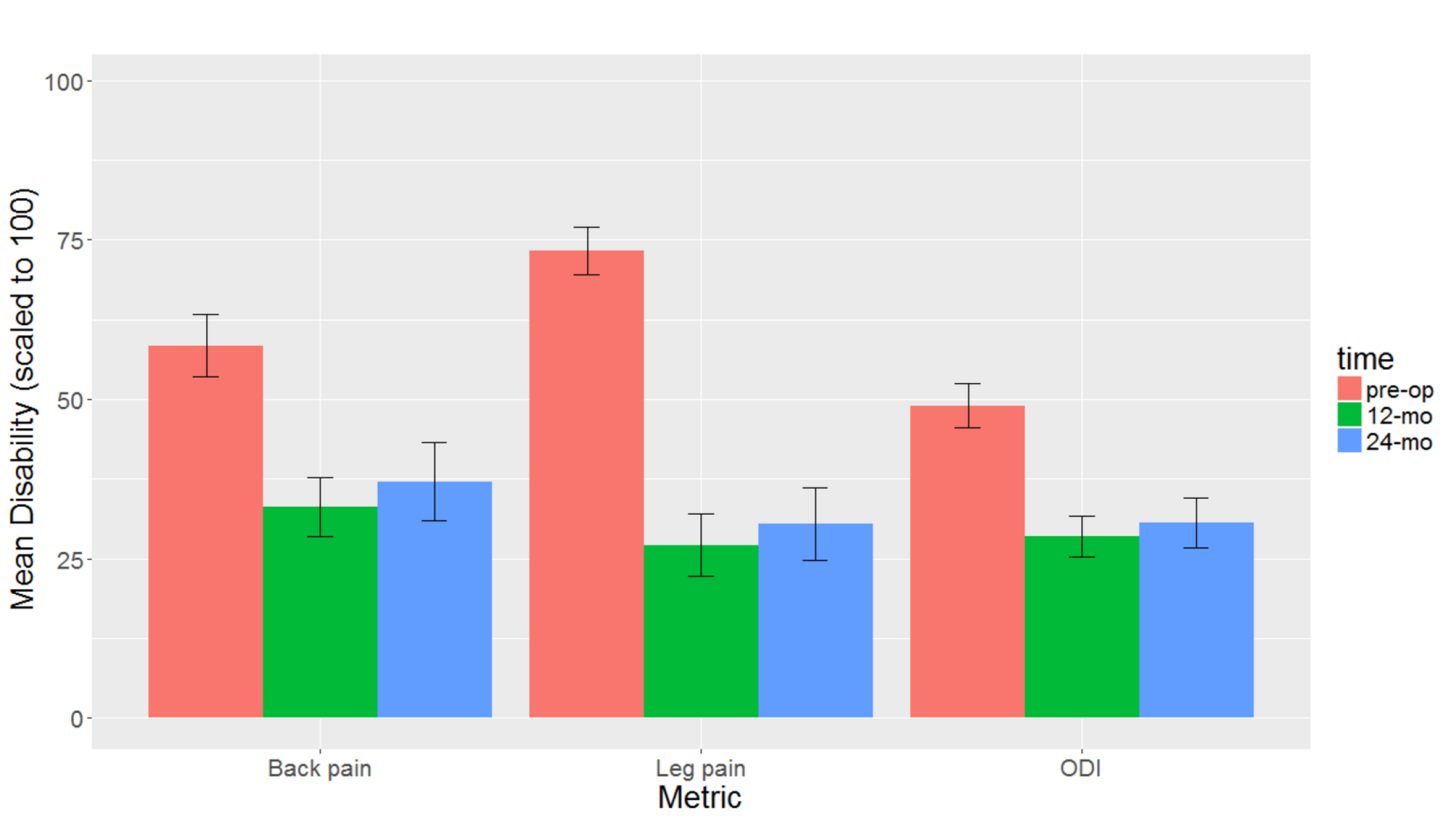
Back pain, leg pain, and Oswestry Disability Index vs. time.

Back pain (VAS 5.84 to 3.32, p<0.001), leg pain (VAS 7.33 to 2.71, p<0.001), and ODI (48.97 to 28.50, p<0.001) demonstrated statistically significant improvements at the 12-month follow-up. Back pain (VAS 3.71, p=0.004), leg pain (VAS 3.04, p<0.001), and ODI (30.63, p<0.001) improvements were maintained at 24 months. No outcomes deteriorated between the 12-month and the 24-month follow-up (p>0.050; Table 2, Figure 3).

**Table 2:** Comparisons of pre-surgery, 12 month, and 24 month follow-up outcomes for FLTD cases. *: p<0.05, FLTD: far lateral tubular decompression, ODI: Oswestry Disability Index, SD: standard deviation

Variable	Pre-surgery		12 month follow-up		24 month follow-up		pre - 12	pre - 24	12 - 24
	Mean ± SD	n	Mean ± SD	n	Mean ± SD	n	p-value	p-value	p-value
Back Pain	5.84 ± 2.94	38	3.32 ± 2.81	38	3.71 ± 2.93	24	<0.001*	0.004*	0.694
Leg Pain	7.33 ± 2.29	39	2.71 ± 2.99	38	3.04 ± 2.76	24	<0.001*	<0.001*	0.796
ODI	48.97 ± 20.92	37	28.50 ± 19.71	38	30.63 ± 18.45	24	<0.001*	<0.001*	0.732

Four patients (9.5%) required subsequent open revision decompression and fusion. These four patients underwent isolated FLTD during the first year of the study investigation, with dates of surgery ranging from December 2015 to May 2016. No patients since then have required subsequent open revision. The subsequent open revisions were performed at a median of 9.5 months after index microdecompression (range two months - 24 months). Three of the four patients had complex spine pathologies (i.e. occult pars fracture) and a history of prior spine surgery (i.e. emergency decompression for cauda equina syndrome, four-level cervical fusion). The fourth patient had 15° of lumbar spine scoliosis and required a multi-level decompression.

Six patients (17.6%) required a subsequent minimally invasive procedure after isolated FLTD. These cases were not considered failures, as patients acknowledged and accepted that this may be warranted. Moreover, these patients generally experienced initial relief, with symptoms recurring months or even years later. Subsequent microdecompression was performed at a median of 17 months after index microdecompression (range seven months - 24 months). Three of the six patients exhibited pathology recurring at the same level and side but inside the canal at the lateral recess rather than outside the foramen. One patient was an elderly patient with multiple prior surgeries who did not improve clinically. The remaining two patients developed pathological problems at a level different than that of their initial presentation.

Subsequent open revision was associated with prior spine surgery (RR=2.85 (2.07-3.63), p=0.045) and scoliosis ≥10° (RR=6.33 (4.87-7.80), p=0.013; Table 3).

**Table 3:** One-way associations with need for revision of index FLTD cases. *: p<0.05, BMI: body mass index, FLTD: far lateral tubular decompression, p: proportion, SD: standard deviation

Variable	No need for major revision	Subsequent major revision	n	p-value	
	Mean ± SD	Mean ± SD			
	p (%)	p (%)			
	n=38	n=4			
Age (years)	67.89 ± 13.38	63.75 ± 6.70	42	0.493	
Wedge angle (°)	2.10 ± 2.39	2.67 ± 2.89	34	0.636	
Segmental lordosis (°)	21.94 ± 7.80	16.00 ± 7.00	34	0.224	
Operative time (minutes)	90.88 ± 35.43	82.75 ± 22.97	41	0.982	
Length of stay (days)	0.78 ± 1.20	0.56 ± 0.47	42	0.813	
Gender (male)	23/38 (60.5%)	2/4 (50.0%)	42	0.683	
BMI>30	18/36 (50.0%)	1/4 (25.0%)	40	0.342	
Current smoker	3/38 (7.9%)	1/4 (25.0%)	42	0.268	
Diabetes	8/38 (21.1%)	0/4 (0%)	42	0.308	
Synovial cyst	3/38 (7.9%)	0/4 (0%)	42	0.560	
Multiple levels	3/38 (7.9%)	1/4 (25.0%)	42	0.268	
Prior spine surgery	10/38 (26.3%)	3/4 (75.0%)	42	0.045	*
Disc procedure	7/38 (18.4%)	1/4 (25.0%)	42	0.750	
Laminotomy	2/38 (5.3%)	0/4 (0%)	42	0.638	
Spondylolisthesis ≥ 4 mm	6/38 (15.8%)	0/4 (0%)	42	0.391	
Scoliosis ≥ 10°	3/38 (7.9%)	2/4 (50.0%)	42	0.013	*

## Discussion

The present study highlights both the benefits and challenges of utilizing FLTD, a minimally invasive, same-day surgery to treat symptomatic degenerative lumbar foraminal stenosis. Significant reductions in back pain, leg pain, and ODI were demonstrated postoperatively and maintained at two years. The current literature defining minimum clinically important differences (1.2, 1.6, and 12.8 for back pain, leg pain, and ODI, respectively) suggests that the improvement observed in this study is not only statistically significant but also clinically significant [10]. Patients who failed the treatment were successfully treated with facet resection and fusion. Prior spine surgery and scoliosis ≥10° were significantly associated with surgical failure requiring a secondary procedure.

Numerous studies have described paramedian or far-lateral decompression techniques with varying results [4-8]. Kim et al. evaluated extraforaminal decompression without fusion using a paramedian incision coming directly down on the pars interarticularis and compared this technique to posterior lumbar interbody fusion (PLIF) [8]. They reported a revision rate of 12% (3/25 patients) in the decompression group and concluded that the extraforaminal decompression yielded results at least as good as PLIF. Yamada et al. also used a paramedian approach with partial pars resection at the foramen and reported 20% treatment failure at follow-up [5]. Of the failures, 89% had degenerative lumbar scoliosis, which the authors deemed a significant risk factor for treatment failure. In our study, we were able to quantify scoliosis ≥10° as a significant risk factor for surgical failure. Hari et al. described a minimally invasive lateral foraminotomy using tubular retractors with partial lateral facetectomy [11]. None of 12 patients investigated required additional surgery at a follow-up period of at least one year. Chang et al. described a microsurgical foraminotomy via a posterolateral transmuscular approach and a contralateral oblique approach, with excellent or good results by the MacNab Scale in 33 of 39 patients (85%), at a mean follow-up of 25.5 years [12]. Haimoto et al. evaluated 12 far-lateral decompressions at an average follow-up of 19 months and showed a revision rate of 42% (5/12 patients) [4]. Risk factors for failure included an increased preoperative coronal plane disc wedge angle of the L5-S1 segment (3.5 degrees versus 1.1 degrees in the success group). Cho et al. described an open far-lateral, muscle-splitting approach to L5-S1 in 21 patients [7]. At an average of 18 months' follow-up, 33% failed to demonstrate clinical improvement. Treatment failure was associated with higher segmental lordosis angles in a neutral upright posture (18.4° vs. 13°, p=0.02). Unlike Haimoto and Cho, we did not see an association between coronal wedge and segmental lordosis angles.

From a clinical standpoint, foraminal stenosis remains a challenging problem to address, particularly utilizing minimally invasive techniques. Continued movement of the segment with disc bulging and facet hypertrophy may well contribute to the recurrence of symptoms. In a registry study of MIS transforaminal decompressions by Sclafani et al., the authors reported a revision rate of 2% for those undergoing discectomy and 28% for those undergoing decompression from bony foraminal stenosis [6]. Furthermore, patients who underwent a discectomy demonstrated a significant improvement in ODI (19.4 points, p=0.0002) while those with bony foraminal stenosis only improved by 7.1 points (p=0.06). Bony foraminal stenosis from low-grade spondylolisthesis requires adequate intraoperative decompression without compromising facet joint stability. These technical challenges may have contributed to the differences seen in clinical outcomes in the study by Sclafani et al. [6]. In the present study, the need for simultaneous discectomy (8/42) or the presence of a low-grade spondylolisthesis (6/42) was not significantly associated with adverse outcomes.

The statistical significance of the VAS and ODI score improvements is likely reflective of a clinical significance, with the ODI minimum clinically important difference estimated to range between 2.92 to 15.36, depending on the calculation method [10]. Chung et al. described the current trends of defining clinical improvements in spine surgery and highlighted the need for future studies to better define the “clinical importance” of a change in patient-reported outcomes [13]. Taking into account the current improvement criteria, this approach appears to provide patients the option to significantly alleviate pain and regain functionality with a minimally invasive, same-day surgery. As the trend to value-based healthcare system continues to evolve, discussing the limitations of this technique, as well as addressing realistic expectations, is important to optimize patient satisfaction.

This case series is limited by the small sample size. Large database studies aside, this is characteristic of studies investigating minimally invasive decompressions. A control group was not included for analysis. Ideally, a non-surgical group would serve as a control, however, this has the potential to compromise patient care and may introduce selection bias to the study. As such, this study aimed to describe outcomes at one-year and two-year follow-up rather than to demonstrate superiority to non-operative management or other surgical techniques. Furthermore, all procedures were performed by a single surgeon; however, we believe the technique is reproducible, and that this limitation does not affect the generalizability of the results. A strength of this study is the prospective nature of the data collection, which allows for the identification of predictors of failure using a novel technique.

## Conclusions

In patients with symptomatic degenerative lumbar foraminal stenosis who underwent FLTD of the lumbar neuroforamen, back pain, leg pain, and disability scores showed consistent improvement at two years. Based on our reported outcomes and complications, prior spine surgery and scoliosis ≥ 10° may be considered relative contraindications to this technique. Continued outcomes collection at five years postoperatively will better describe the long-term outcomes of this approach.
